# Expression Profile of Long Noncoding RNAs in Human Earlobe Keloids: A Microarray Analysis

**DOI:** 10.1155/2016/5893481

**Published:** 2016-12-22

**Authors:** Liang Guo, Kai Xu, Hongbo Yan, Haifeng Feng, Linlin Chai, Guozheng Xu

**Affiliations:** ^1^Department of Plastic Surgery, Wuhan General Hospital of Guangzhou Military Command of Chinese PLA, Wuhan, Hubei, China; ^2^Department of Plastic and Reconstructive Surgery, Southwestern Hospital, Third Military Medical University, Chongqing, China; ^3^Department of Neurosurgery, Wuhan General Hospital of Guangzhou Military Command of Chinese PLA, Wuhan, Hubei, China

## Abstract

*Background*. Long noncoding RNAs (lncRNAs) play key roles in a wide range of biological processes and their deregulation results in human disease, including keloids. Earlobe keloid is a type of pathological skin scar, and the molecular pathogenesis of this disease remains largely unknown.* Methods*. In this study, microarray analysis was used to determine the expression profiles of lncRNAs and mRNAs between 3 pairs of earlobe keloid and normal specimens. Gene Ontology (GO) categories and Kyoto Encyclopedia of Genes and Genomes (KEGG) pathway enrichment analyses were performed to identify the main functions of the differentially expressed genes and earlobe keloid-related pathways.* Results*. A total of 2068 lncRNAs and 1511 mRNAs were differentially expressed between earlobe keloid and normal tissues. Among them, 1290 lncRNAs and 1092 mRNAs were upregulated, and 778 lncRNAs and 419 mRNAs were downregulated. Pathway analysis revealed that 24 pathways were correlated to the upregulated transcripts, while 11 pathways were associated with the downregulated transcripts.* Conclusion*. We characterized the expression profiles of lncRNA and mRNA in earlobe keloids and suggest that lncRNAs may serve as diagnostic biomarkers for the therapy of earlobe keloid.

## 1. Introduction

Keloids are defined as pathologically formed scars that exceed the boundary of the original wound [1]. They are also deemed as benign dermal tumors that are unique to humans. Etiologically, keloids may occur because of minor skin injury, such as body piercing and insect bites. In addition, it is widely agreed that the incidence rate of keloid is significantly higher in populations with darker skin, such as Africans and Asians. The external ear is one of the most common sites for keloid formation [2]. Many different treatment modalities such as surgical excision, intralesional corticosteroids, radiotherapy, and pressure earrings have been used for keloids [3, 4]. Although it has unclear etiology, the development of keloid could be considered as a process of abnormal wound healing, during which redundant extracellular collagen fibers as well as proteoglycans are deposited [5]. It is known that various molecular factors contribute to this process, for example, growth factors [6, 7], cytokines [8], and related gene pathways [9]. Some among them may be the key points that could stop or reverse this pathologic process. For example, transforming growth factor-*β* (TGF-*β*) receptor was recently reported to be a potential target in treating keloid [10]. However, deeper understanding of the molecular mechanism of keloid formation is still required for detecting critical biological factors and for the further development of effective therapies.

It is known that 90% of the human genome is transcribed to RNAs that do not code proteins (noncoding RNAs). A lot of evidence suggests that long noncoding RNA (lncRNA; >200 nucleotides) regulates protein-coding genes at the transcriptional and posttranscriptional levels, as well as transcription control [11, 12]. It is known that lncRNAs play important roles in cellular differentiation, development, and disease [11, 12]. However, for earlobe keloids, the expression or function of lncRNAs has not been studied to date.

It the present study, global expression profiles of the lncRNAs and the mRNAs from 3 pairs of earlobe keloid specimens and normal skin tissues were detected using a microarray technique, from which significantly dysregulated lncRNAs and mRNAs were screened. These results indicated that the aberrant expression levels of lncRNAs may have important roles in the development of earlobe keloid and that knowing the differently expressed lncRNAs might provide useful biomarkers for earlobe keloid therapy and diagnosis.

## 2. Materials and Methods

### 2.1. Patients and Specimens

The study procedures were approved by the Ethics Review Board of Wuhan General Hospital of Guangzhou Military Command of the People's Liberation Army and it was carried out in accordance with the Declaration of Helsinki (2008) of the World Medical Association. Keloid was diagnosed by the overgrowth of a scar that obviously exceeded the boundary of the original wound. Demographic and clinical characteristics of the patients were extracted from their medical records. Earlobe keloid specimens were obtained from the resected keloid at our outpatient clinic. The normal skin specimens were obtained from the ear of the same patient. All patients were fully informed of the aim and protocol of the study and gave written informed consent to participate in the study.

### 2.2. RNA Isolation, Quantification, and Quality Control

Total RNA was extracted with the mirVana miRNA Isolation Kit (Applied Biosystems) and then eluted with 100 mL of nuclease-free water. Total RNA was quantified using a NanoDrop ND-2000 spectrophotometer (Thermo Fisher Scientific) and the integrity of RNA was determined using an Agilent 2100 bioanalyzer and RNA 6000 Nano Kit (Agilent Technologies).

### 2.3. RNA Labeling and Array Hybridization

RNA sample preparation and microarray hybridization were performed according to Agilent One-Color Microarray-Based Gene Expression Analysis Protocol (Agilent Technologies, Santa Clara, CA, USA) with minor modifications. RNA was purified from 100 *μ*g total RNA after removal of rRNA using RNeasy Mini Kit (Qiagen). After that, specimens were amplified and transcribed into cRNA, and cyanine-3-CTP was applied to label the cRNA (Quick Amp Labeling Kit: One-Color; Agilent). Labeled cRNA was once again purified with the RNeasy Mini Kit (Qiagen) and quantified using a NanoDrop ND-2000 spectrophotometer (Thermo Fisher Scientific).

The cRNA was fragmented and hybridized using an Agilent Gene Expression Hybridization Kit (Agilent): 0.6 *μ*g labeled cRNA was fragmented by adding 5.0 *μ*L 10x blocking agent and 1.0 *μ*L 25x fragmentation buffer, and then the mixture was heated at 60°C for 30 minutes. After that, 25 *μ*L 2x GEx Hybridization Buffer was added to stop the fragmentation reaction. Finally, 50 mL hybridization solution was dispensed into the gasket slide and assembled to the lncRNA expression microarray slide. The slides were incubated for 17 hours at 65°C in an Agilent Hybridization Oven. The hybridized arrays were washed, fixed, and scanned with using the Agilent DNA Microarray Scanner (part number, G2505C).

### 2.4. Data Analysis

Data were extracted with Agilent Feature Extraction software 11.7.1.1. GeneSpring GX 12.5 (Agilent Technologies) was used to normalize the quantiles of the raw data. The lncRNAs are carefully constructed using the quality-controlled, public transcriptome databases (RefSeq, UCSC Known Genes, lncRNAWiki, LNCipedia, NONCODE v4, fRNAdb v3.4, Broad lincRNA, GENCODE, etc.), as well as landmark publications. After that, lncRNAs and mRNAs with significant differential expression between the two groups were identified, and the volcano plot was drawn. Hierarchical clustering was performed using MeV 4.9.0 (http://www.tm4.org/mev.html), and heat maps were obtained by this analysis. Gene Ontology (GO) analysis was performed based on Gene Ontology (www. geneontology.org), which provided three structured networks of defined terms that describe gene product functions. Kyoto Encyclopedia of Genes and Genomes (KEGG, http://www.genome.jp/kegg/) database was used for pathway analysis of the differentially expressed genes.

### 2.5. Quantitative Real-Time PCR (qRT-PCR)

The total RNA was isolated using mirVana miRNA Isolation Kit (Applied Biosystems) and was then reverse-transcribed using PrimeScript RT reagent kit with gDNA Eraser (Perfect Real Time; TaKaRa). The expression of five upregulated lncRNAs and five downregulated lncRNAs was measured by qRT-PCR using SYBR Green assays (TaKaRa), and GAPDH was used as an internal control. The expression level of each lncRNA was represented as a fold change using 2^−ΔΔCt^ methods. The expression levels of lncRNAs differentially expressed between earlobe keloid specimens and normal skin specimens were analyzed using Student's *t*-test with SPSS version 17.0 [13].

### 2.6. Statistics

Statistical analysis was performed with SPSS version 19.0. The differences in expression levels of tested lncRNAs and mRNAs between earlobe keloid and normal skin tissues were assessed using Student's *t*-test, and fold change ≥ 2.0 and *P* < 0.05 were considered significant. Fisher's exact test was used for GO analysis and KEGG pathway analysis. *P* < 0.05 was considered significant.

## 3. Results

### 3.1. Differentially Expressed lncRNAs

**Figure 1:**
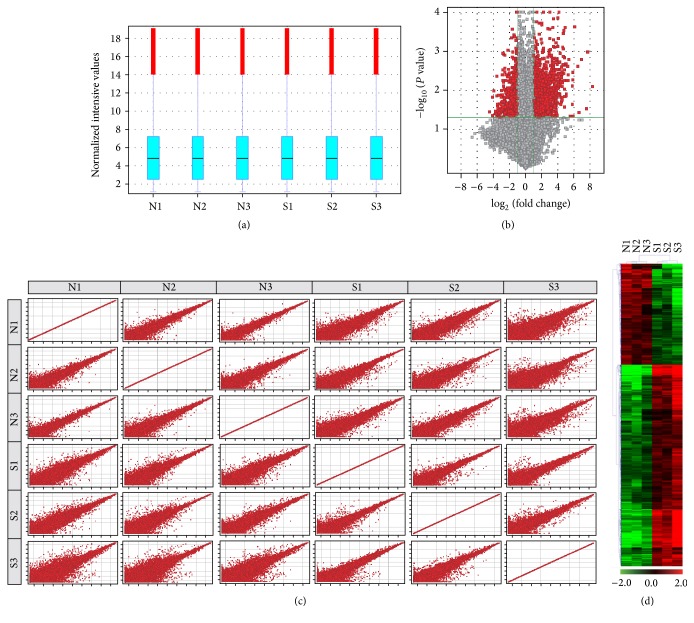
Expression profiles of lncRNAs in earlobe keloid and normal skin specimens. (a) Box-whisker plots of lncRNAs showed the distributions of intensities from all samples. (b) Volcano plots showed variation in lncRNA expression. The vertical lines correspond to 2.0-fold upregulation and downregulation and the horizontal line represents a *P* value of 0.05. (c) Scatter plots show variation in lncRNA expression. (d) Hierarchical clustering shows lncRNA expression profiling. Cluster analysis arranges samples into groups based on their expression levels, which allows us to hypothesize the relationships among samples. “Red” indicates highly relative expression, and “green” indicates lowly relative expression.

The baseline data for the 3 patients (3 pairs of specimens) included in the study are shown in Table 1. In order to compare the distributions of intensities from all samples, we used a box plot to visualize the distributions of a dataset. Box-whisker plotting suggested similar distribution of the data from six RNA gene chips (Figure 1(a)). The expression profiles of 2068 lncRNAs indicated that they were differentially expressed (fold change ≥ 2.0 and *P* < 0.05) between earlobe keloid specimens and normal skin specimens (shown in the lncRNA profiling). Variations in lncRNA expression among specimens were shown by volcano plotting and scatter plotting (Figures 1(b) and 1(c)). Among these lncRNAs, 1290 were upregulated more than twofold in the earlobe keloid specimens compared to the normal skin specimens, while 778 lncRNAs were downregulated more than twofold. lncRNA expression data are deposited at Gene Expression Omnibus under accession number GSE83286. The top 20 differentially expressed lncRNAs are listed in Tables 2 and 3. Finally, to infer the relationships among specimens, hierarchical clustering was performed to show distinguishable lncRNA expression patterns among samples (Figure 1(d)).

### 3.2. Differentially Expressed mRNAs

**Figure 2:**
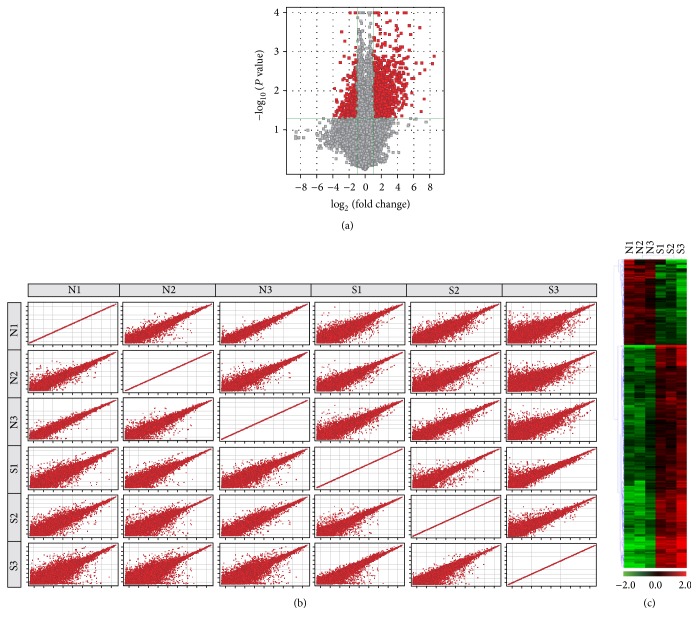
Expression profiles of mRNAs in earlobe keloid and normal skin specimens. (a and b) Volcano and scatter plots show differences in expression. The vertical green lines delimit 2.0-fold upregulation and downregulation. Red plots represent mRNAs with >2.0-fold change and corrected *P* value < 0.05. (c) Hierarchical clustering shows mRNA expression profiling. Cluster analysis arranges samples into groups based on their expression levels, which allows us to hypothesize the relationships among samples. “Red” indicates highly relative expression, and “green” indicates lowly relative expression.

A total of 1511 mRNAs were differentially expressed between the two tissues (fold change ≥ 2.0 and *P* < 0.05). A total of 1092 of 1511 mRNAs were expressed significantly higher in earlobe keloid specimens and 419 mRNAs were expressed significantly lower compared to normal skin specimens (shown in the mRNA profiling). mRNA expression data are deposited at Gene Expression Omnibus under accession number GSE83286. The top 20 differentially expressed mRNAs are listed in Supplemental Tables  1 and  2 in Supplementary Material available online at http://dx.doi.org/10.1155/2016/5893481. Variations in the mRNA expression among specimens were shown by volcano plotting and scatter plotting (Figures 2(a) and 2(b)). Hierarchical clustering showed that mRNA expression modes among samples were distinguishable (Figure 2(c)).

### 3.3. GO Analysis

**Figure 3:**
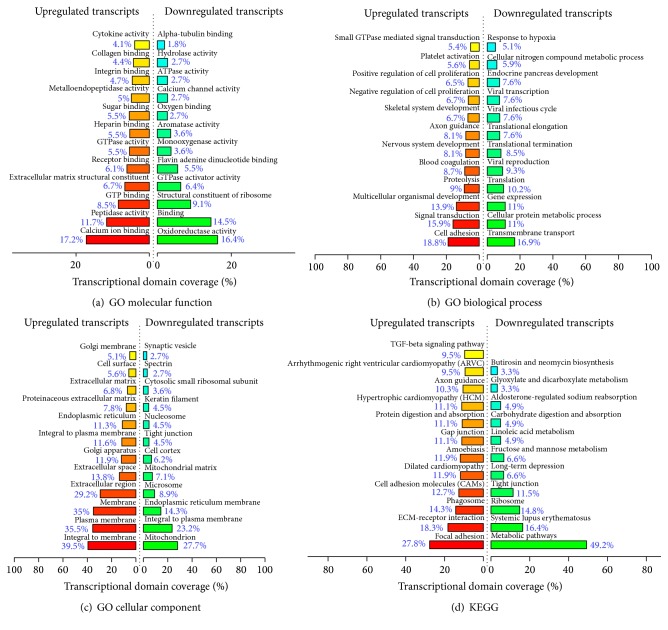
(a) Top 12 enriched GO terms for differentially expressed mRNAs for molecular function. The bar plot shows the transcriptional domain coverage. (b) Top 12 enriched GO terms for differentially expressed mRNAs for biological processes. The bar plot shows the transcriptional domain coverage. (c) Top 12 enriched GO terms for differentially expressed mRNAs for cellular components. The bar plot shows the transcriptional domain coverage. (d) The results of KEGG pathway enrichment analysis. The bar plot shows the transcriptional domain coverage of the enrichment pathway.

The GO project is a collaborative effort to construct and use ontologies to facilitate the biologically meaningful annotation of genes and their products in a wide variety of organisms [14]. We performed GO analysis for lncRNAs to determine molecular function, biological processes, and cellular components. For molecular function (Figure 3(a)), calcium ion binding (GO:0005509) had the highest transcriptional domain coverage (TDC, 17.2%) in upregulated transcripts, while oxidoreductase activity (GO:0016491; TDC, 16.4%) was highest in downregulated transcripts. In biological processes (Figure 3(b)), it was found that upregulated genes were enriched most in the process of cell adhesion (GO:0007155; TDC, 18.8%). In contrast, downregulated genes were enriched most in the process of transmembrane transport (GO:0007155; TDC, 16.9%). In the cellular components (Figure 3(c)), it was detected that integral to membrane (GO:0016021) had the highest enrichment of upregulated genes (TDC, 39.5%), and mitochondria had the highest enrichment of downregulated genes (GO:0005886; TDC, 27.7%).

### 3.4. KEGG Analysis

KEGG pathway enrichment analysis was used for differentially expressed genes to identify pathways represented among the lncRNAs identified in the earlobe keloid gene expression signature. KEGG analysis suggested that 24 pathways were significantly correlated with upregulated gene expression. The focal adhesion pathway had the highest enrichment of increased transcription (TDC, 27.8%) and comprised 35 targets genes. Pathway analysis also revealed that 11 pathways corresponded to downregulated transcripts and that the most enriched network was metabolic pathways (TDC, 49.2%), which comprised 30 target genes (Figure 3(d)). Many of these pathways are reported to be associated with keloid, including the gene category focal adhesion pathway [15], TGF-*β* signaling pathway [16–18], mitogen-activated protein kinase (MAPK) pathway [19], and gap junction pathway [20, 21].

### 3.5. QRT-PCR Validation

**Figure 4:**
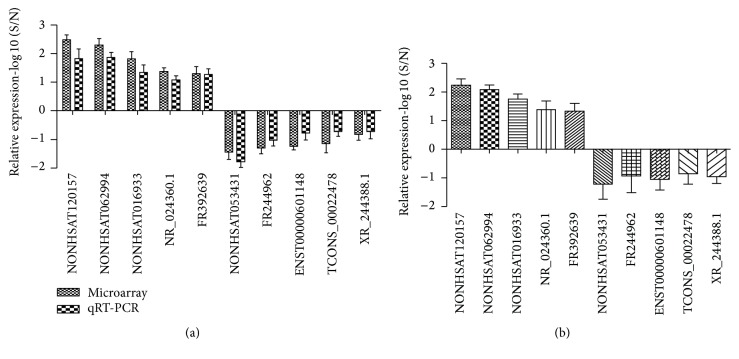
Quantitative RT-PCR validation of 10 differentially expressed lncRNAs. (a) Comparison of fold change [log_10_⁡(S/N)] of lncRNAs between microarray and quantitative RT-PCR results (S: earlobe keloid specimens; N: normal skin specimens). (b) Relative expression levels of lncRNAs in 10 other pairs of earlobe keloid and normal skin specimens (*P* < 0.05).

To verify the microarray data, five upregulated lncRNAs (NONHSAT120157, NONHSAT062994, NONHSAT016933, NR_024360.1, and FR39263) and five downregulated lncRNAs (NONHSAT053431, FR244962, ENST00000601148, TCONS_00022478, and XR_244388.1) were randomly selected from the differentially expressed lncRNAs. We detected the expression levels of these lncRNAs in 10 earlobe keloids tissues and normal skin samples (Supplemental Table 3) using qRT-PCR. As shown in Figure 4, the qRT-PCR results and microarray data are consistent.

## 4. Discussion

Emerging evidence shows that a set of noncoding RNAs (for example, miRNA) is involved in the mechanism of keloid formation [22–25]. lncRNAs are larger than miRNAs and have more complex structure. Deregulated expression of lncRNA disrupts cellular physiology and then leads to pathology [26–28]. Thus, we suggest that lncRNAs may play crucial roles in many biological processes and are vital to the formation of earlobe keloids. However, the profile and biological function of lncRNAs for earlobe keloid remain largely unknown. Thus, in the present study, we established the expression profile of lncRNAs in human earlobe keloids.

We analyzed lncRNA and mRNA expression profiles in the tissues of earlobe keloid and control tissues to reveal the potential roles of lncRNAs in the pathogenesis of earlobe keloid. High-throughput microarray techniques uncovered differential expression between 3 pairs of earlobe keloid and normal skin specimens. We identified that 1290 lncRNAs and 1092 mRNAs were upregulated and 778 lncRNAs and 419 mRNAs were downregulated in all 3 earlobe keloid and normal tissues (fold change ≥ 2.0, *P* < 0.05). GO and KEGG pathway analysis were used to explore the possible biological functions and potential mechanisms of lncRNAs and mRNAs in earlobe keloids. In fact, Liang and colleagues have previously identified differential expression of lncRNAs and mRNAs between 3 pairs of keloid and normal skin tissue by microarray (32). Compared with their results, our study has several differences. First, tissues used here were earlobe keloid and normal specimens, and the expression profiles of lncRNAs were significantly different from the previous results. Second, to verify the microarray data, the expression levels of five upregulated lncRNAs and five downregulated lncRNAs were detected in 10 earlobe keloids tissues and normal skin samples using qRT-PCR, and the results were consistent with microarray data.

An integrative method including pathway was developed to identify possible functional relationships between the different RNA molecules. Based on the differentially expressed mRNAs, pathway analysis revealed which biological functions and mechanisms were involved in earlobe keloid formation. Our results suggest that different biological processes, such as cell-cell adhesion, cell migration, cell death, cell junction formation, epithelial to mesenchymal transition (EMT), TGF-*β*, and MAPK, are among the significantly enriched mRNAs. Most of these pathways are involved in the process of tissue fibrosis. For example, studies in a wide range of experimental models have revealed that TGF-*β* is a central mediator of keloid fibrogenesis. It is reported that Loureirin B attenuated the contraction of fibroblasts which was induced by TGF-*β* in hypertrophic scar formation (33). Yan et al. have reported that EMT plays crucial roles in keloid formation [29]. Gobin et al. have shown that emodin-loaded liposomes decrease survival rate of keloids which express high levels of receptor tyrosine kinase (RTK) (included in the focal adhesion pathway) [30]. Among these related pathways, we found that focal adhesion, extracellular matrix receptor interaction, cell adhesion molecules, and gap junction-associated pathway showed significant changes in upregulated and downregulated mRNAs. For example, 35 differentially expressed mRNAs were involved in the focal adhesion pathway and 16 differentially expressed mRNAs were enriched in cell adhesion molecules. Otherwise, 7 of which were downregulated in the tight junction related pathway. These results indicated that cell adhesion and tight associated signaling may play an important role in the mechanism of earlobe keloid formation, which is not identified by other researches.

Our study used microarray data to analyze systematically and comprehensively differentially expressed lncRNAs and mRNAs between normal skin and earlobe keloid tissues. Many differentially expressed lncRNAs could play a vital role in regulating earlobe keloid formation through various pathways. In our present study, we found that TGF-*β*, MAPK, cell tight, and adhesion related signaling series mRNAs may interact with IncRNAs. Previous reports demonstrated that ADAM proteins are involved in cell adhesion, cell fusion, cell signaling, and proteolysis. ADAM33 is a member of ADAM family that is associated with keloid scars in the northeastern Chinese population (36). ADAM12 are reluctant to adhere to fibronectin, a key ECM protein in keloids (37). Patients suffering from collagen VI related myopathies caused by mutations in COL6A1, COL6A2, and COL6A3 often also display skin abnormalities, like formation of keloids or “cigarette paper” scars, dry skin, striae rubrae, and keratosis pilaris (follicular keratosis) (38). Keloid fibroblasts were propagated in culture and their proliferative behaviour and response to the epidermal growth factor (EGF) were studied (39). Our results found that NONHSAT016934, NONHSAT016933, NONHSAT016928, and NONHSAT077639 expressions were increased, whereas NONHSAT097800 expression was decreased in earlobe keloid and normal tissues. These lncRNAs were associated with the related genes of keloid (ADAM12, COL6A3, and EGF). We will carry out further studies of these differentially expressed lncRNAs to establish their functions in earlobe keloid formation.

In conclusion, we studied the differential expression profile of lncRNAs and mRNAs in earlobe keloid and normal skin tissues. Our microarray analysis indicated that lncRNAs are involved in the pathological process of earlobe keloid formation. Therefore, subgroup analysis of lncRNAs should be performed to explore this relationship in the future. In addition, we will select numbers of samples to deepen the research into the lncRNA molecular mechanism and biochemical function in order to provide a novel accurate method for therapy of earlobe keloid.

## Supplementary Material

The top 20 up- and down-regulated mRNAs.

## Figures and Tables

**Table 1:** Baseline data of included patients.

Case	Age (years)	Gender	Reason of skin injury	Size of keloid (cm × cm × cm)	History of keloid (months)
1	21	Female	Earlobe piercing	2.0 × 1.3 × 0.8	10
2	34	Female	Earlobe piercing	1.8 × 1.5 × 1.2	14
3	24	Female	Earlobe piercing	2.7 × 2.0 × 1.5	17

**Table 2:** The top 20 upregulated lncRNAs.

Seq. name	Source	Fold change	Chrom.	Strad.	txStrat	txEnd	Associated gene name
NONHSAT120157	NONCODE v4	302.566	chr7	−	37946864	37949441	SFRP4
NONHSAT062994	NONCODE v4	198.76	chr19	−	18896928	18897844	COMP
ENST00000424523	Ensembl	187.8763	chr7	+	92484223	92546465	
NONHSAT016934	NONCODE v4	121.1942	chr10	−	127823937	127843874	ADAM12
NONHSAG007229	NONCODE v4	98.20973	chr10	−	134634754	134637851	TTC40
NONHSAT135001	NONCODE v4	85.73119	chr9	+	131745793	131747541	NUP188
NONHSAT016933	NONCODE v4	66.81899	chr10	−	127779305	127798357	ADAM12
NONHSAT033754	NONCODE v4	59.65379	chr13	+	50191636	50192101	
NONHSAT102388	NONCODE v4	57.30576	chr5	+	79377827	79379011	THBS4
NONHSAT016928	NONCODE v4	56.89644	chr10	−	127700956	127703336	ADAM12
NONHSAT076769	NONCODE v4	55.34357	chr2	+	216476099	216669548	LINC00607
NONHSAT033252	NONCODE v4	45.90168	chr13	−	38137358	38144948	POSTN
NONHSAG013256	NONCODE v4	45.0158	chr13	−	38136835	38145672	POSTN
ENST00000557618	Ensembl	40.71083	chr14	+	60981837	61021634	
ENST00000597626	Ensembl	37.49051	chr21	+	35287852	35341659	
NONHSAG030448	NONCODE v4	36.29881	chr2	−	216232403	216237205	FN1
NONHSAT056875	NONCODE v4	34.17572	chr18	+	907552	909671	ADCYAP1
NONHSAG052055	NONCODE v4	32.53344	chr9	+	34084331	34096676	DCAF12
NONHSAT100815	NONCODE v4	31.91256	chr5	+	28524293	28602803	
NONHSAT077639	NONCODE v4	30.05823	chr2	−	238241611	238243429	COL6A3

**Table 3:** The top 20 downregulated lncRNAs.

Seq. name	Source	Fold change	Chrom.	Strad.	txStrat	txEnd	Associated gene name
NONHSAT053431	NONCODE v4	22.78803	chr17	+	37395854	37400623	FBXL20
NONHSAT030286	NONCODE v4	17.52123	chr12	+	101988749	102021958	MYBPC1
FR244962	fRNAdb v3.4	16.8388	chr7	+	31551811	31552010	
ENST00000601148	Ensembl	15.31924	chr19	+	51843949	51847370	
TCONS_l2_00026076	Broad lincRNA	13.31326	chr7	+	80804833	80828289	
FR193036	fRNAdb v3.4	12.53563	chr19	−	56526608	56527152	
NONHSAT125631	NONCODE v4	12.31555	chr8	+	25398695	25408293	
TCONS_l2_00016248	Broad lincRNA	12.02637	chr20	+	37230676	37256614	
NONHSAT030224	NONCODE v4	12.02533	chr12	−	100560001	100562998	GOLGA2P5
NONHSAT076673	NONCODE v4	11.6689	chr2	−	211074313	211081443	ACADL
NONHSAT137402	NONCODE v4	11.66708	chrX	+	69454505	69457167	AWAT1
ENST00000580420	Ensembl	11.61363	chr18	+	29522538	29524119	
FR174595	fRNAdb v3.4	11.10483	chr6	+	118888744	118889041	
NONHSAT077942	NONCODE v4	11.03293	chr2	−	242455829	242457154	
NONHSAT060814	NONCODE v4	10.87916	chr19	+	7410710	7411049	
NONHSAT097800	NONCODE v4	10.56074	chr4	+	110897243	110898692	EGF
NONHSAT070090	NONCODE v4	10.32572	chr2	+	36991437	36993016	VIT
TCONS_00022478	Broad lincRNA	10.21703	chr14	+	38205181	38208450	
ENST00000556024	Ensembl	9.802968	chr14	−	38025363	38036300	
NONHSAT102735	NONCODE v4	9.773005	chr5	+	90142207	90144638	GPR98
