# Supplementary material for: Expression Profile of Long Noncoding RNAs in Human Earlobe Keloids: A Microarray Analysis
Source: Biomed Res Int. 2016 Dec 22;2016:5893481. doi: 10.1155/2016/5893481 (PMC5215475; doi:10.1155/2016/5893481)
Supplement: Supplementary file 1 — The top 20 up- and down-regulated mRNAs. [file 5893481.f1.docx]

Supplemental Table 1. The top 20 up-regulated mRNAs.

| GeneSymbol | Sourse | Fold change | chrom | strad | txStrat | txEnd |
| --- | --- | --- | --- | --- | --- | --- |
| FNDC1 | Agilent_humanG3V2 | 339.45508 | chr6 | + | 159692704 | 159692763 |
| ASPN | Agilent_humanG3V2 | 287.45502 | chr9 | _ | 95218993 | 95218934 |
| ASPN | Agilent_humanG3V2 | 160.34145 | chr9 | _ | 95227261 | 95227202 |
| CILP2 | Agilent_humanG3V2 | 146.07954 | chr19 | + | 19657135 | 19657194 |
| FNDC1 | Agilent_humanG3V2 | 119.5518 | chr6 | + | 159646694 | 159646753 |
| ZIM2 | Agilent_humanG3V2 | 112.908646 | chr19 | _ | 57286046 | 57285987 |
| COMP | Agilent_humanG3V2 | 104.85319 | chr19 | _ | 18893877 | 18893726 |
| SFRP4 | Agilent_humanG3V2 | 103.55692 | chr7 | _ | 37945829 | 37945770 |
| SFRP4 | Agilent_humanG3V2 | 76.37584 | chr7 | _ | 37945565 | 37945534 |
| THBS4 | Agilent_humanG3V2 | 60.038475 | chr5 | + | 79378905 | 79378964 |
| LRRC15 | Agilent_humanG3V2 | 52.817978 | chr3 | _ | 194076556 | 194076497 |
| P4HA3 | Agilent_humanG3V2 | 52.037197 | chr11 | _ | 73980697 | 73979228 |
| NPTX2 | Agilent_humanG3V2 | 51.492733 | chr7 | + | 98258890 | 98258949 |
| COL5A2 | Agilent_humanG3V2 | 45.57008 | chr2 | _ | 189897488 | 189897429 |
| COL5A1 | Agilent_humanG3V2 | 42.161182 | chr9 | + | 137734063 | 137734122 |
| COL1A1 | Agilent_humanG3V2 | 41.817833 | chr17 | _ | 48261568 | 48261509 |
| LUM | Agilent_humanG3V2 | 37.94903 | chr12 | _ | 91497705 | 91497646 |
| SFRP2 | Agilent_humanG3V2 | 37.691116 | chr4 | _ | 154702884 | 154702825 |
| GRIN2D | Agilent_humanG3V2 | 34.89015 | chr19 | + | 48948128 | 48948187 |
| CTHRC1 | Agilent_humanG3V2 | 33.643726 | chr8 | + | 104390268 | 104390327 |

Supplemental Table 2. The top 20 down-regulated mRNAs.

| GeneSymbol | Sourse | Fold change | chrom | strad | txStrat | txEnd |
| --- | --- | --- | --- | --- | --- | --- |
| CHRM1 | Agilent_humanG3V2 | 13.778804 | chr11 | _ | 62676214 | 62676155 |
| KRT75 | Agilent_humanG3V2 | 13.082559 | chr12 | _ | 52822420 | 52822220 |
| CARD18 | Agilent_humanG3V2 | 12.783865 | chr11 | _ | 105009673 | 105009614 |
| CCDC129 | Agilent_humanG3V2 | 12.1413765 | chr7 | + | 31696057 | 31696116 |
| C2orf72 | Agilent_humanG3V2 | 11.661879 | chr2 | + | 231914355 | 231914414 |
| RAB26 | Agilent_humanG3V2 | 11.24166 | chr16 | + | 2204081 | 2204140 |
| DEFB1 | Agilent_humanG3V2 | 10.767873 | chr8 | _ | 6728240 | 6728181 |
| KRTAP12-2 | Agilent_humanG3V2 | 10.417324 | chr21 | _ | 46086571 | 46086512 |
| ABCC6 | Agilent_humanG3V2 | 10.141947 | chr16 | _ | 16315206 | 16315147 |
| S100A2 | Agilent_humanG3V2 | 9.127293 | chr1 | _ | 153533890 | 153533831 |
| CCDC129 | Agilent_humanG3V2 | 8.917011 | chr7 | + | 31692445 | 31692504 |
| SEC14L6 | Agilent_humanG3V2 | 8.844951 | chr22 | _ | 30921043 | 30920984 |
| AWAT1 | Agilent_humanG3V2 | 8.274515 | chrX | + | 69460042 | 69460101 |
| HNF1A | Agilent_humanG3V2 | 8.119339 | chr12 | + | 121435710 | 121435769 |
| PANK1 | Agilent_humanG3V2 | 7.8431005 | chr10 | _ | 91342846 | 91342787 |
| LMO3 | Agilent_humanG3V2 | 7.801019 | chr12 | _ | 16701693 | 16701634 |
| WNK2 | Agilent_humanG3V2 | 7.6507287 | chr9 | + | 96080578 | 96080637 |
| CYP4F8 | Agilent_humanG3V2 | 13.778804 | chr19 | + | 15739621 | 15740029 |
| PRR22 | Agilent_humanG3V2 | 13.082559 | chr19 | _ | 5783222 | 5783163 |
| PLEKHH1 | Agilent_humanG3V2 | 12.783865 | chr14 | + | 68055609 | 68055668 |

Supplemental Table3. Baseline data of included patients.

| Case | Age (years) | Gender | Reason of skin injury | Size of keloid (cm×cm×cm) | History of keloid (months) |  |
| --- | --- | --- | --- | --- | --- | --- |
| 1 | 25 | Female | Earlobe piercing | 2.0×1.3×0.8 | 10 |  |
| 2 | 34 | Female | Earlobe piercing | 1.8×1.5×1.2 | 14 |  |
| 3  4  5  6  7  8  9  10 | 24  21  35  27  17  20  26  37 | Female  Female  Female  Female  Female  Female  Female  Female | Earlobe piercing  Earlobe piercing  Earlobe piercing  Earlobe piercing  Earlobe piercing  Earlobe piercing  Earlobe piercing  Earlobe piercing | 2.7×2.0×1.5  1.6×1.6×0.6  2.3×1.8×1.4  1.8×1.3×1.5  1.2×1.3×0.8  1.8×1.3×1.4  2.5×1.3×1.6  2.8×1.6×1.8 | 17  8  23  13  6  14  16  26 |  |
